# Occurrence of the invasive Spanish slug in gardens: can a citizen science approach help deciphering underlying factors?

**DOI:** 10.1186/s12898-018-0179-7

**Published:** 2018-08-02

**Authors:** Daniel Dörler, Matthias Kropf, Gregor Laaha, Johann G. Zaller

**Affiliations:** 1 Institute of Zoology, University of Natural Resources and Life Sciences, Vienna, Vienna, Austria; 2 Institute for Integrative Nature Conservation Research, University of Natural Resources and Life Sciences, Vienna, Vienna, Austria; 3 Institute of Applied Statistics and Computing, University of Natural Resources and Life Sciences, Vienna, Vienna, Austria

**Keywords:** *Arion vulgaris*, *Arion lusitanicus*, Participation, Pest slugs, Weather, Neobiota, DNA barcoding

## Abstract

**Background:**

The Spanish slug (*Arion vulgaris,* also known as *A. lusitanicus*) is considered one of the most invasive species in agriculture, horticulture and private gardens all over Europe. Although this slug has been problematic for decades, there is still not much known about its occurrence across private gardens and the underlying meteorological and ecological factors. One reason for this knowledge gap is the limited access of researchers to private gardens. Here we used a citizen science approach to overcome this obstacle and examined whether the occurrence of *Arionidae* in Austrian gardens was associated with meteorological (air temperature, precipitation, global solar radiation, relative humidity) or ecological factors (plant diversity, earthworm activity). Occurrence of the invasive *A. vulgaris* versus the similar-looking native *A. rufus* was compared using a DNA-barcoding approach.

**Results:**

Slugs were collected from 1061 gardens from the dry Pannonian lowland to the wet alpine climate (altitudinal range 742 m). Slug abundance in gardens was best explained and negatively associated with the parameters “sum of the mean air temperature in spring”, “number of frost days in the previous winter” and “mean daily global solar radiation on the day of data collection”. Precipitation, plant diversity and earthworm activity were also related to slug abundance, but positively. Out of our genetic sampling of collected slugs, 92% belonged to *A. vulgaris*.

**Conclusions:**

Our study showed that citizen science (i) is a feasible approach to record species occurrence in restricted areas across a wide geographical range and (ii) could be more widely employed in order to identify underlying environmental factors of species occurrence.

## Background

Invasive alien species have been recognized as a threat to biodiversity, human health and economy by many countries [1]. Through global trade routes organisms are transported both deliberately and accidentally around the globe, sometimes finding a new home in areas they never could have reached on their own. In many cases these species remain unnoticed until they establish viable populations, spread quickly and outcompete native species, or become pestiferous in agriculture, horticulture or private gardens. At this stage residents of areas where the dispersal occurs are often among the first to notice this change in their environment [2, 3]. As a consequence, many environmental protection agencies, research facilities and municipalities use citizen science as a method of choice to detect invasive species at early stages [4, 5]. Citizen science describes a method where citizens and scientists work together in scientific projects. The citizens usually collect data, analyze data or are even involved in the whole scientific process [6].

In the current study we tested the citizen science approach to assess the occurrence of the Spanish slug *Arion vulgaris* Moquin-Tandon 1855 (formerly known as *A. lusitanicus* auct. non-Mabille) in private gardens in Austria. Urban biota can host a variety of native and non-native species [7] and gardens are particularly interesting areas, because they are ‘designed’ by humans [8] and therefore species numbers often vary from one garden to the next. In Austria *A. vulgaris* was described for the first time in 1971 in a village near Vienna [9]. The origin of this slug is still controversial [10, 11], but it is nevertheless listed among the 100 most invasive species in Europe [12]. In the meanwhile it has become one of the most common pest slugs in private gardens, horticulture and agriculture in central Europe [13–15]. The Spanish slug feeds on many vegetables and ornamentals as well as dead plant material [16, 17] and can survive low temperatures and droughts [18, 19]. Under normal circumstances, most Spanish slugs die after oviposition at the end of the year [20], but can live up to 3 years if they are unable to breed [21]. They can adapt to a variety of environmental factors and therefore compete with native slug species [22]. The activity of *A. vulgaris* is strongly correlated to humidity [19, 23, 24] and temperature [18, 21]. Additionally, the time of day [25, 26] and the availability of suitable shelters affect slug activity [25, 27]. Some studies also observed that earthworms [28, 29] or plant diversity [30] affected the extent of herbivory by slugs.

In many areas, *A. vulgaris* co-occurs with two similar looking native *Arion* species (the red slug *A. rufus* and the black slug *A. ater*). Recent studies have shown that no clear mating barrier exists between these three species and that fertile hybrids occur [31–34]. This makes traditional species identification difficult and genetic analyses a useful addition [35, 36].

Hence, the goals of the current study was (I) to test a citizen science approach in order to determine the occurrence and abundance of slugs in particular across gardens in Austria, (II) to determine any association of slug abundance with meteorological factors, plant diversity and earthworm activity in these gardens, and (III) to investigate the occurrence of the invasive *A. vulgaris* versus the native *A. rufus* by using DNA sequencing.

## Methods

### Study area and data collection

This study focused on private gardens across Austria. These gardens were distributed across all Austrian federal states, but most of them were located in the highly urbanized regions of Vienna and the greater Vienna area. Although the study covers a broad range of geographical conditions, there is more weight to the landscapes of the Vienna region: hilly terrains and flatlands with meadows, forests and cultivated areas. We chose a citizen science approach to independently assess the abundance and occurrence of slugs in Austrian gardens in the spring seasons 2014 and 2015. In 2014 the assessment took place from the 2nd of April to the 12th of June, in 2015 from the 3rd of April to the 15th of May. Participants were undergraduate students at the University of Natural Resources and Life Sciences, Vienna. All participants got 1 h training explaining the goal of this project and the sampling protocol. This method allowed us to get data on slug and plant diversity from private properties. To enable people to collect scientifically sound data, we designed a simple protocol. The participants used a cardboard quadrat (20 × 20 cm) that they randomly placed in their garden. This quadrat served as an artificial shelter for slugs during the day. No baits where used to attract slugs. The quadrats were left undisturbed on the same spot for 2 days, after which the slugs under the quadrats where counted, photographed and collected. Additionally, data were collected on altitude of the collection site, on the number of plant species within a radius of 2 m around the quadrat, number of earthworm casts under the quadrat as a measure for earthworm activity [37] and previous and current control methods against molluscs employed in the garden.

In spring 2014 participants used the smartphone app Epicollect and Epicollect+, respectively [38]. Epicollect and Epicollect+ are open source smartphone applications available for Android and iOS for data collection in the field. The collected data were stored in a central database and can be exported in various formats for further analyses. In our study participants used the apps to submit pictures of slugs and data on location, altitude, number of plant species, number of earthworm casts and slug management methods. In 2015 the participants entered the data for the same parameters in a spreadsheet (Microsoft Excel) and uploaded it together with the associated photographs at the university’s online learning platform based on Moodle (https://learn.boku.ac.at/). The number of slugs was deduced from the spreadsheet and the photographs uploaded via app or on the Moodle platform, respectively, in the year 2015. In 2014 slugs were collected by the participants and preserved in 80% ethanol for further inspection by us. Slug numbers were then obtained by counting of the preserved slugs. All data were validated using the photographs the participants sent or the slug samples, respectively. Inconsistent or incomplete data sets were discarded and not included in further analyses.

In addition, we procured data on weather conditions from the nearest weather stations from each location of the year before the collection and the collection year from Austrian Federal meteorological and geophysical data provider (Zentralanstalt für Meteorologie und Geodynamik ZAMG). From these data we extracted various measures for vapour pressure (hPa), air temperature (°C), relative air humidity (%), precipitation (mm) and global radiation (J/cm^2^) from the collection day and periods before collection. See Table 1 for more details on the parameters considered for statistical analysis.

**Table 1:** List of abbreviations and corresponding parameters that were taken into account in the statistical analysis

Parameter abbreviation	Description
Abiotic parameters	
alt	Meters above sea level
cfc	Applied slug control methods in the year previous to data collection
cc	Applied slug control methods in the year of data collection
comc	Applied slug control methods in the previous year and the year of data collection
Biotic parameters	
slugs	Number of slugs under the cardboard
worms	Number of earthworm casts under the cardboard
plants	Number of plants in a radius of 2 m around the data collection point
Meteorological parameters from the data collection year	
mvp	Mean vapour pressure
matemp	Mean air temperature
maxatemp	Maximum air temperature
minatemp	Minimum air temperature
mrhumid	Mean relative humidity
prec	Precipitation
rad	Global radiation on the day of data collection
smetcy	Sum of the mean daily temperature from the beginning of the year until the day of data collection
smaxtcy	Sum of the maximum daily temperature from the beginning of the year until the day of data collection
smintcy	Sum of the minimum daily temperature from the beginning of the year until the day of data collection
spreccy	Sum of the precipitation from the beginning of the year until the day of data collection
frostcy	Number of days below 0 °C from January to April
spscy	Sum of the precipitation in March (the month directly before sampling) of the year of data collection
mpscy	Mean precipitation in March (ditto)
Meteorological data from the year previous to data collection	
smetpy	Sum of the mean temperature
smaxtpy	Sum of the maximum temperature
smintpy	Sum of the minimum temperature
sprecpy	Sum of the precipitation
frostpy	Number of days below 0 °C from September to December
drypy	Number of days without precipitation from March to September
spspy	Sum of precipitation from March to September
frostwint	Number of days below 0 °C from the winter before data collection (September to April)

### DNA barcoding approach

To identify and discriminate the invasive slug *A. vulgaris* from other slug species we used a DNA barcoding approach on 127 slug garden samples representing different altitudes and regions within Austria. The gardens were selected to cover all altitudinal levels and provinces represented in the whole data set.

DNA was extracted using the protocol of DNeasy^®^ Blood & Tissue Kit, following the manufacturer’s protocol (Qiagen, Hilden, Germany). Incubation time was usually 1 h at 56 °C. Genomic DNA extracts were stored at − 20 °C until processing. The DNA barcode region of the cytochrome C oxidase subunit 1 (COI) gene (cf. [39]) was amplified using the LCO-1490 forward and the HCO-2198 reverse primers [40]. Amplification was performed by taking 1 µl of genomic DNA extract and adding 16.3 µl double distilled H_2_O, 2.5 µl PCR Buffer, 2.5 µl MgCl_2_ (25 mM), 0.5 µl dNTP (2 mM), 0.5 µl of either forward or reverse primer (10 pmol/µl), 1 µl BSA and 0.2 µl Taq (5 U/µl). We used the following PCR protocol: initial step of 4 min at 94 °C, 5 cycles of 94 °C, 60 s/44 °C, 90 s/72 °C, 90 s, followed by 30 cycles of 94 °C, 60 s/49 °C, 75 s/72 °C, 75 s, and a post-treatment of 7 min at 72 °C. In order to check whether the DNA extraction and PCR amplification was successful, we run agarose gel electrophoresis. When the results of the gel electrophoresis were not convincing, we repeated DNA extraction and the amplification step and ran agarose gel electrophoresis again. Final cycle sequencing was processed by LGC Genomics (Berlin, Germany).

After a pre-check running the NCBI BLAST search algorithm (https://blast.ncbi.nlm.nih.gov/Blast.cgi?PROGRAM=blastn&PAGE_TYPE=BlastSearch&LINK_LOC=blasthome), we used eleven published reference DNA sequences of five different *Arion* species [41–44] and of the grey garden slug *Deroceras reticulatum* [45] for slug identification. At first, all sequences (n = 138) were manually aligned using BioEdit 7.2.5 [46]. Subsequently, genetic distances among individual slug samples were quantified by using the K2P model [47], as is commonly done in non-phylogenetic, but species-identification DNA barcoding analysis [cf. 48]. The genetic distance matrix was used to generate a neighbor-joining (NJ) tree [49] with bootstrap support (BS > 60%) values indicated above species branches based on 10,000 replicates. These analyses were all performed running PAUP* (version 4.0b10; [50]). Different DNA sequences of the mitochondrial COI marker obtained in our analysis were deposited in the ‘European Nucleotide Archive’ under the accession numbers LS974172-LS974197.

### Ecological statistics

All statistical analyses were done using R (Version 3.0.1) and R Studio (Version 1.0.136) [51]. In order to identify which factors are strongly correlated we first calculated a heatmap using Spearman rank correlations with the R package “stats”. The resulting heatmap displays the strength of the relationships of the correlation matrix in colour codes and additionally groups the factors that are intercorrelated. Therefore, it is possible to visually identify factors that are rather independent from each other and have a combined effect on the response variable [52]. This makes it possible to select most important factors based on the results of former studies on slug ecology and on the current results of our heatmap.

In a next step we used the R package MASS to calculate a generalized linear model and a stepwise forward and backward Poisson regression based on the identified factors from the heatmap and compared the Akaike Information Criteria (AIC) of both models to identify the one with the better fit. To account for potential multicollinearity we also calculated the variance inflation factors (VIF).

## Results

### Ecological data

**Fig. 1:**
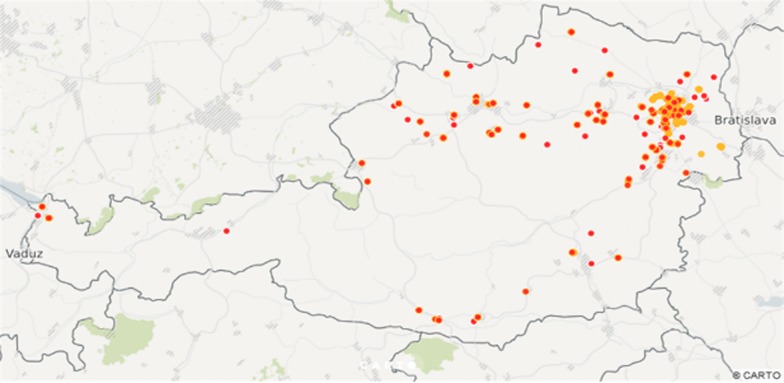
Map of garden collection points across Austria. Yellow dots represent data from 2014, red dots data from 2015. Map created with free version of carto (https://carto.com/)

After consolidation of the received samples from citizen scientists, we obtained 1061 garden data sets, comprised of 687 in 2014 and 374 in 2015, sometimes including more than one slug, in 2014 and 2015, respectively (Fig. 1). In 459 of the 1061 data sets (43%) slug control measures were applied either in the year of data collection or in the year before.

The altitudinal range of the observations was from 134 to 876 m a.s.l. Altogether 1269 slugs were analysed, with a mean of 1.2 ± 2.5 SD (standard deviation) slugs and a maximum of 20 slugs under one sampling quadrat. On average, 3.9 ± 3.3 SD plant species were found within a radius of 2 m around the quadrat; the maximum was 31 different plant species within this area in one garden. Earthworm activity measured as the number of earthworm casts directly under the quadrat was 0.7 ± 1.6 SD earthworm casts with a maximum of 19 casts under one quadrat in one garden.

**Fig. 2:**
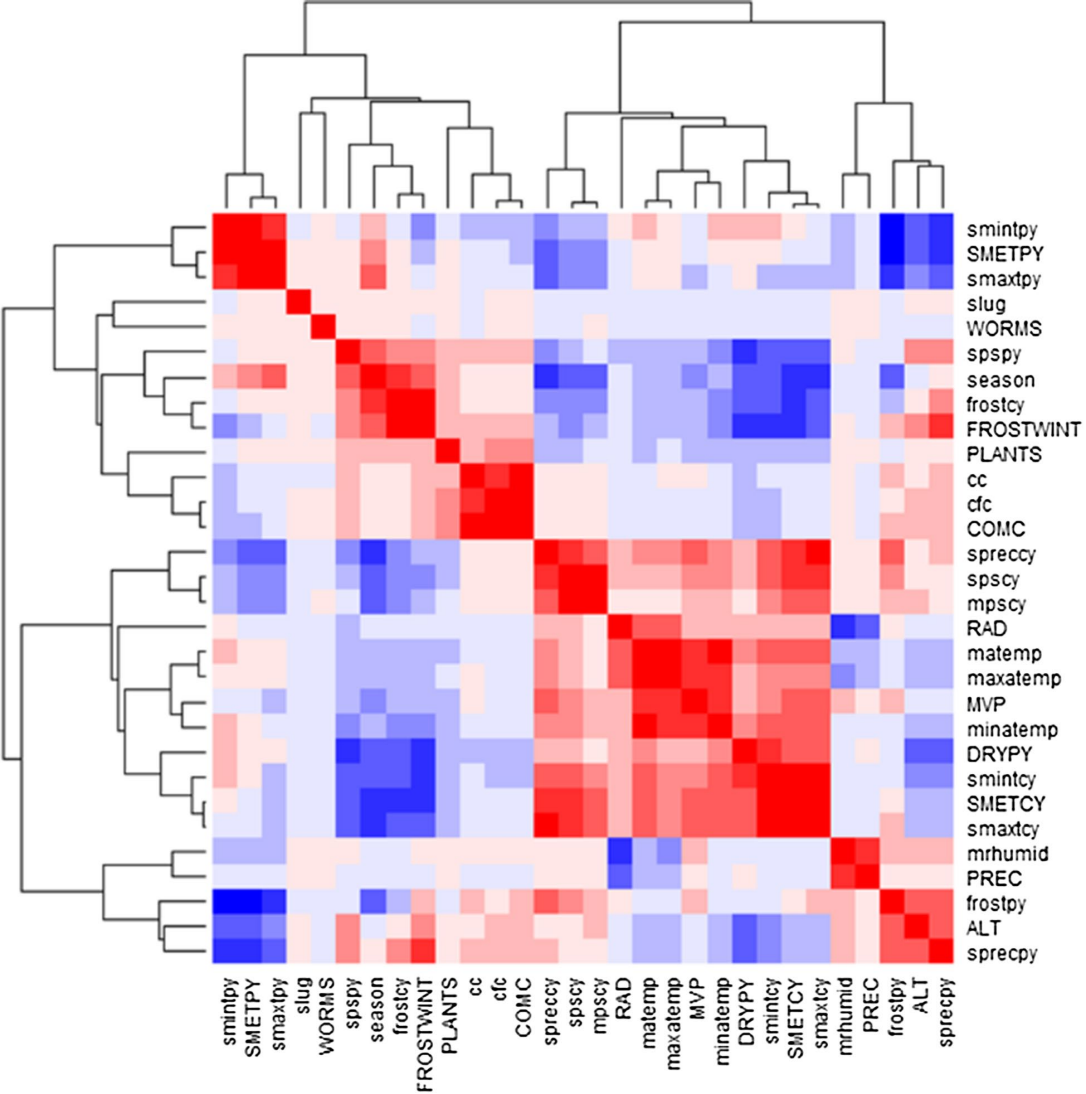
Heatmap of correlations between slug numbers and meteorological and ecological factors using Spearman correlations. Different shades of blue (negative correlation) and red (positive correlation) show the strength of the correlations; the more intensive the colours the stronger the correlations. Parameters in capital letters were used for further statistical analyses. Please refer to Table 1 for full names of the parameters. Map created with R (Version 3.0.1) and R Studio (Version 1.0.136)

Heatmaps representing correlation of slug abundance and possible influencing factors are presented in Fig. 2. Groups of intercorrelated parameters are visible along the main diagonal. Overall, six different parameter groups may be distinguished. Out of these six groups we chose the sum of the mean temperature from the year previous to data collection (smetpy), the number of days below 0 °C from the winter before data collection (September to April; frostwint), the applied slug control methods in the previous year and the year of data collection (comc), the global radiation on the day of data collection (rad), the mean vapour pressure on the day of data collection (mvp), the number of days without precipitation from March to September from the year previous to data collection (drypy), the sum of the mean daily temperature from the beginning of the year until the day of data collection (smetcy), the precipitation on the day of data collection (prec) and the meters above sea level of the sampling site (alt) and additionally the number of plants in a radius of 2 m around the data collection point (plants) and the number of earthworm casts under the cardboard (worms) for further analyses (Table 1). We chose these parameters because on the one hand they represent their respective groups and on the other hand they are often found to affect slugs in the literature. Individual correlations with slug abundance were generally low.

To test the combined effect of these parameters on slug number we employed a generalized linear model (Table 2). A negative correlation was found between the number of slugs and the sum of the mean daily temperature from the beginning of the year until the day of data collection (smetcy), the number of days below 0 °C from the winter before data collection (September to April; frostwint), the global radiation on the day of data collection (rad), the mean vapour pressure on the day of data collection (mvp), the number of days without precipitation from March to September from the year previous to data collection (drypy), the sum of the mean temperature from the year previous to data collection (smetpy) and the meters above sea level of the sampling site (alt). A positive correlation was found with the number of earthworm casts under the cardboard (worms), the applied slug control methods in the previous year and the year of data collection (comc), the precipitation on the day of data collection (prec) and the number of plants in a radius of 2 m around the data collection point (plants). All variance inflation factors stayed sufficiently low (using a threshold value of 5 as a rule of thumb), so multicollinearity was not an issue. We checked the model for redundant information by computing a stepwise parameter fit. Based on AIC the stepwise regression model did not perform better (AIC for both models was 4308.1), therefore we chose the generalized linear model fitted to the full set of eleven parameters selected from the heatmap.

**Table 2:** Relationship between the number of slugs in gardens and meteorological and ecological factors

Coefficients	Estimate	Std. error	z value	Pr(> |z|)	Significance
(Intercept)	5.52E+00	8.43E−01	6.541	6.11E−11	***
Sum of the mean daily temperature from the beginning of the year until the day of data collection (smetcy)	− 6.51E−04	1.67E−04	− 3.888	0.000101	***
Number of earthworm casts under the cardboard (worms)	7.71E−02	1.37E−02	5.64	1.70E−08	***
Number of days below 0 °C from the winter before data collection (September to April) (frostwint)	− 8.69E−03	2.22E−03	− 3.91	9.22E−05	***
Applied slug control methods in the previous year and the year of data collection (comc)	8.02E−02	3.37E−02	2.381	0.017267	*
Global radiation on the day of data collection (rad)	− 2.75E−04	4.48E−05	− 6.123	9.17E−10	***
Mean vapour pressure on the day of data collection (mvp)	− 2.85E−02	1.14E−02	− 2.494	0.012639	*
Number of days without precipitation from March to September from the year previous to data collection (drypy)	− 1.66E−02	4.07E−03	− 4.08	4.50E−05	***
Sum of the mean temperature from the year previous to data collection (smetpy)	− 3.91E−04	9.62E−05	− 4.066	4.79E−05	***
Precipitation on the day of data collection (prec)	7.78E−03	3.87E−03	2.012	0.044244	*
Meters above sea level of the samplings site (alt)	− 1.31E−03	3.32E−04	− 3.937	8.27E−05	***
Number of plants in a radius of 2 m around the data collection point (plants)	5.22E−02	7.05E−03	7.404	1.32E−13	***

Relationship between the number of slugs in gardens and meteorological and ecological factors, calculated with a generalized linear model. Abbreviations are explained in Table 1

Significance codes are *** p < 0.001, ** p < 0.01, * p < 0.05

### Genetic data

**Fig. 3:**
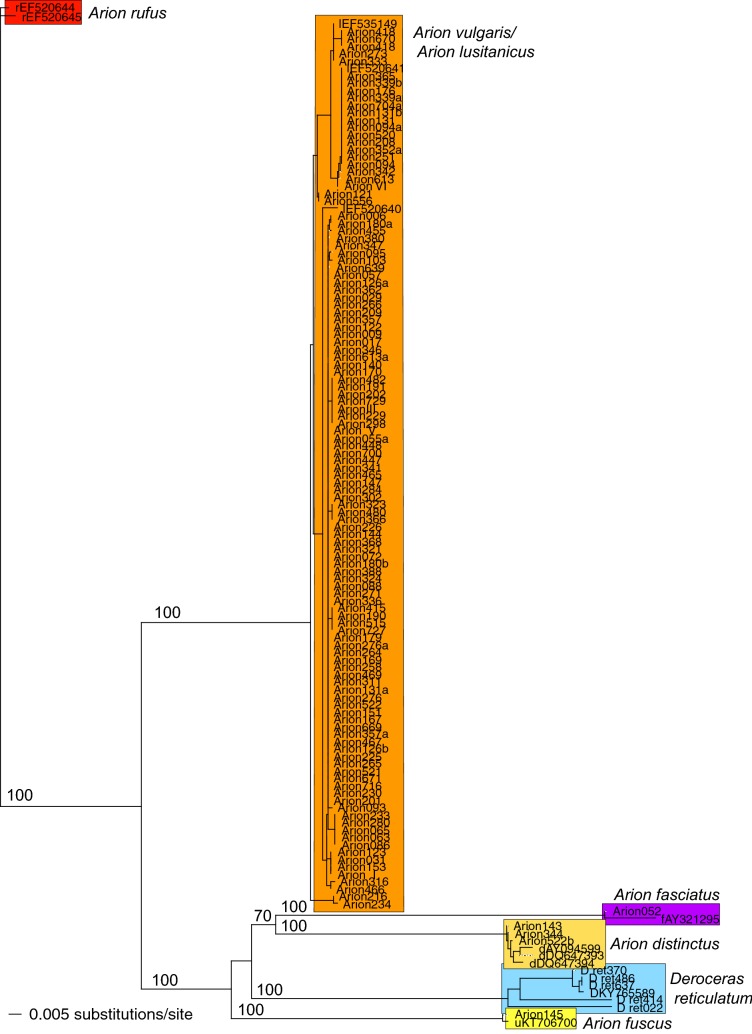
Neighbour-Joining tree based on K2P genetic distances displaying 138 individual samples. Already published DNA sequences are indicated by their GenBank accession numbers. Our samples are simply numbered (e.g. Arion_001). Abbreviated with “r” and in red are the two reference COI barcodes for *A. rufus* [53], abbreviated with “l” and in orange are all barcodes of *A. vulgaris* (reference barcodes still named *A. lusitanicus;* 53), abbreviated with “d” and in dark yellow *A. distinctus* [43], abbreviated with “u” and in light yellow *A. fuscus* [44], abbreviated with “f” and in violet *A. fasciatus* [42], and abbreviated with “D” and in blue is *D. reticulatum* [45]

The length of the alignment of 138 individual slug COI sequences was 638 base pairs. The tree topology is displayed as a NJ phylogram based on K2P genetic distances between these samples (Fig. 3).

Our 127 newly generated individual slug sequences are representative of five different species: *Arion vulgaris*, *A. fuscus*, *A. distinctus*, *A. fasciatus* and *Deroceras reticulatum*. However, most of our samples (92%) represent *A. vulgaris*; and further species, like for instance the native *A. rufus*, were not detected within our data set. All species-branches had 100% bootstrap support indicating unambiguous species identification of slugs using this DNA barcoding approach. Grouping of individual sequences reflecting altitudinal and/or geographical clusters was not observed (i.e. genotypes observed are randomly distributed across Austria).

## Discussion

Our results indicate that meteorological parameters and plant diversity have a significant impact on the abundance of slugs in Austrian gardens. This confirms general results found in previous studies also on the level of individual gardens and indicates that citizen science is a suitable method for assessing the abundance of slugs on private properties.

In general, the use of a citizen science approach made it possible to collect data on a large geographical scale, covering all Austrian provinces in only 3 months. Additionally, we could get access to data from private properties, which we could not get otherwise. The project potentially raised the participants’ awareness for invasive species and ecosystem processes, although we did not measure this. The trade-off we had to accept was that we could not collect as extensive data as in a conventional project, in order to not overstrain participants and to keep motivation high.

We found a significant positive correlation between number of slugs observed and precipitation on collection day (prec), suggesting a high slug activity during moister conditions [24, 25, 54]. The significant negative association between slug abundance and mean vapour pressure (mvp) is connected to the vapour pressure deficit, the difference between the actual vapour pressure and the vapour pressure the air would have, when it is saturated with moisture, which has been described as one main factor affecting slug activity [19, 24].

The finding that the sum of the mean daily temperature from the sampling year (smetcy), the sum of the daily mean temperature of the year previous to data collection (smetpy) and the number of frost days from the previous winter (frostwint) influence slug abundance in gardens also supports the results of previous studies [18, 21, 23, 55], although in some respects other than expected:

The negative correlation between the number of slugs and the temperature sum of the sampling year (smetcy) and the year previous to data collection (smetpy) contradicts findings of colleagues, who demonstrated higher slug activity with rising temperatures [24, 56]. There could be multiple possible explanations for this finding.

Temperatures under the artificial slug shelters could have risen above tolerable limits, which made the slugs leave their shelter [57] seeking a cooler, shaded refuge under vegetation. Hommay et al. [23] recommended therefore inspections of slug shelters in the early morning before temperatures rise. Although we used cardboard quadrats that do not heat up very fast this could be a (partial) explanation for these results, since we did not check for the time when participants investigated the shelters. We therefore recommend including collection day time in future studies using a citizen science approach.

Another possible explanation could be that temperature is connected to global radiation (rad), which is also negatively correlated with slug numbers in our study and was reported to also affect slug activity [31, 58]. Moreover, in the time of late spring and early summer the temperatures can rise above 30 °C especially in Eastern Austria. As most of the data were collected in this region during rather warm temperatures the lower slug numbers could be the result of suboptimal temperatures for slug development [20, 21, 59]. High temperatures often are connected to drought, a parameter (drypy) correlated significantly negative to slug abundance in our study.

Another parameter in our results that negatively affected slug abundance was winter temperature (frostwint) or more specifically the number of frost days. Organisms have generally two strategies to overwinter: freeze avoidance or freeze tolerance. Some slug species, like *A. vulgaris*, have been shown to have a tolerance for frost [18, 60], others seek frost-free shelters. Particularly in gardens, slugs find good shelter in compost heaps or near heated buildings. However, the number of frost days in winter nevertheless seems to be an important parameter even in gardens, which could also be connected to the parameter altitude (alt), since at higher altitudes winters are usually harsher and we found a negative correlation between altitude and slug abundance.

Moreover, our analysis revealed that general slug abundance was positively correlated with plant diversity (plants) suggesting support of the “more individuals”-hypothesis [61] that states that basically more plant species result in higher productivity and therefore more herbivores. It is also likely that a higher diversity in gardens is associated with a higher richness in structures and potential shelters for slugs, which have been identified as key factor for slug activity, reproduction and survival [25, 62]. Particularly, big slugs are reliant on shelter in the vegetation, because they hardly find shelter in the soil [63]. Additionally, higher plant species richness provides more diverse food sources and more shade for slugs, which could also have an effect on slug numbers.

Something that looked rather contradicting on first sight was the rise in the number of slugs when slug control methods were applied (as showed before, in 43% of the datasets slug control measures have been applied). This does not necessarily mean that control methods did not work properly, but maybe that people who experience more slug damage in their gardens were also more willing to apply control methods. However, this interpretation would need more research in the future.

Our current finding that slug numbers are positively correlated with earthworm activity (worms) suggests that earthworms’ effects on soil structure and moisture [64] benefits slugs in gardens. This seems to contradict other studies reporting that slug herbivory was lower when earthworms were active [28, 29]. However, those studies showed that earthworms stimulated the production of anti-herbivore compounds in the plants which could still take place in the gardens we investigated. Other explanations could be that slugs and earthworms prefer similar habitats, or that this correlation is a methodological artifact, because slugs could have been drawn due to the cardboard shelter to areas where earthworms already resided.

All these above mentioned factors were associated with slug abundance in gardens, in general. Using the citizen science approach we did not distinguish between slug species. However, our DNA analyses of representative samples showed that almost all slugs were identified as *A. vulgaris* (syn. *A. lusitanicus*), and no native *A. rufus* was among them. These results suggest that *A. vulgaris* is most likely the predominant slug species in (Austrian) gardens [65, 66]. Since in recent years more and more hybrids between the invasive *A. vulgaris* and the native *A. rufus* from Northern Europe and Germany have been reported [31–33] and individuals are difficult to distinguish, the use of DNA barcoding approaches seem to be helpful as additional identification tool, the more so as citizens prefer removal of slugs from the gardens and even juvenile individuals could be determined, exactly.

## Conclusions

Although gardens are ‘designed’ by humans [8], our results indicate that meteorological factors like temperature or precipitation are important when studying slug abundance. Because our finding about the role of human affected parameters such as plant diversity on slug abundance contradicts other findings [29], further studies on the role of plant diversity on slug abundance would help to shed some light on these interactions. Additionally, interactions between earthworms and slug herbivory need to be investigated further in the field. Although previous studies showed negative effects of earthworms on slug herbivory in greenhouse experiments, our results indicate a positive relation between earthworm activity and slug abundance, which could also be a result of slugs being drawn to our artificial shelters. Accurate species identification may be difficult in a citizen science project and therefore genetic testing can help to overcome this issue. In summary, our study showed that a citizen science approach can help in gathering data on species occurrences from areas with restricted access (private properties) and can also be used to investigate relationships between species occurrences and meteorological as well as ecological parameters. As citizen science is integrated in early detection programs for invasive alien species by many institutions [4, 5] our results encourage other citizen science projects on early detection and environmental associations.
